# On the Molecular Organ of the Tissues

**Published:** 1852-07

**Authors:** 


*On the molecular origin of the tissues.* By Dr. BENNETT.—The great generalisation of Schwann was that all tissues are derived from cells. Subsequently, it was ascertained that the nucleus, or cell-germ, exercised an influence on the tissues, independent of its cell wall; and it was endeavored to be shown, that some tissues might be derived directly from nuclei. The object of this communication was to point out that the nuclei themselves originated from smaller bodies,—viz., molecules; that these were the origin of every texture, and to indicate some of the laws which governed their formation, arrangement, and subsequent developement. From a review of the observations of Schleiden, Schwann, and Martin Barry, the author pointed out how the first appearance, observable in all developing organisms, was a mass of molecules and granules, which, by aggregating or melting together, constituted the cell-germ. Around the cell-germ other molecules were formed, which again, by melting together, constituted the cell-wall. Further development, in like manner, proceeded by the apposition of molecules. At any period in the process of evolution, the onward progress might be checked when the structure became disintegrated in the inverse manner to its formation: First the cell wall became dissolved, then the nucleus, both of which were reduced, first to molecules then to a fluid. Hence there were molecules of evolution and molecules of disintegration. Occasionally, between the cell wall and nucleus, secondary molecules were formed, which constituted peculiar secretions, as they have been termed. These might be called molecules of transformation.

The author described the origin and mode of formation of these three kinds of molecules, their physiological and pathological importance, and pointed out the advance which had been made in our knowledge of molecular formation by the observations of Ascherson, Harting, and Melsen.

In complex organisms, the higher tissues were formed by an elaboration of blastema, mainly due to the successive evolution, transformation, and disintegration of matter, by means of the different kinds of molecules, of which the author gave numerous examples, derived from the elaboration of the ovum, of the blood, the transformation of insects, the process of fissiparous division in the lower animal forms, etc. He pointed out that molecules had independent movements, sometimes physical, as in the case of Brown's molecular movements, at other times vital, as seen in many organisms. That occasionally we had molecular fibres, from the aggregation end to end of molecules, in the same way as we have nuclear cell fibres. Moreover, each kind of fibre could assume inherent contractility, as in the case of vibriones, which might be called contractile molecular fibres, as spermatozoa might be denominated contractile nuclear, and cilia contractile cell fibres.—*Lond. Monthly Jour.*

